# Comparison Between Inflammatory Biomarkers (High-Sensitivity C-Reactive Protein and Neutrophil-Lymphocyte Ratio) and Psychological Morbidity in Suicide Attempt Survivors Brought to Medicine Emergency

**DOI:** 10.7759/cureus.17459

**Published:** 2021-08-26

**Authors:** Kuldeep Kumar, Shruti Srivastava, Bhanu Sharma, Rajnish K Avasthi, Mrinalini Kotru

**Affiliations:** 1 Medicine, University College of Medical Sciences and Guru Teg Bahadur Hospital, Delhi, IND; 2 Psychiatry, University College of Medical Sciences and Guru Teg Bahadur Hospital, Delhi, IND; 3 Pathology, University College of Medical Sciences and Guru Teg Bahadur Hospital, Delhi, IND

**Keywords:** hscrp, nlr, suicide attempt, psychological morbidity, inflammation

## Abstract

Objectives: This study aimed to compare inflammatory biomarkers (high-sensitivity {hs} C-reactive protein, neutrophil-lymphocyte ratio) and psychological morbidity in suicide attempt survivors.

Methods: One hundred ninety-eight poisoning cases screened, 40 age-matched suicide attempt survivors (SAS), 40 healthy controls (HC) between the age of 18 years and 60 years were included. Complete hemogram, neutrophil-lymphocyte ratio (NLR), hsCRP values obtained, compared with Hospital Anxiety and Depression Scale (HADS), suicide intent scale, presumptive stressful life events scale (PSLES), general health questionnaire 12-item (GHQ-12) (Hindi version), and Hindi Mental State Examination (HMSE).

Results: A statistically significant difference was observed in hsCRP (p=0.016) and NLR (p=0.029) of depressed-suicidal participants vs healthy controls. hsCRP values of anxious-suicidal subjects vs healthy controls showed a statistically significant difference (p=0.001). There was a statistically significant difference between patients, healthy controls in HADS anxiety and HADS depression mean scores (p<0.001). The PSLES items were ranked according to the mean stress scores of all the items (mean±SD), highest four were excessive alcohol use by the family member 47.50 (±27.03), conflicts with in-laws 50 (±27.73), family conflict 50 (±29.42), marital conflict 50.63 (±32.76). There was a statistically significant difference in hemoglobin (p<0.001), red blood cells count (p<0.001), hematocrit (p<0.001) between suicide attempt survivors and healthy controls.

Conclusion: Both hsCRP and NLR have emerged as potential inflammatory biomarkers for depressive patients with suicidal attempts. Additionally, there may be a link between anemia and suicide risk in patients with depression.

## Introduction

Suicide, by definition, is not a disease, but a death that is caused by a self-inflicted intentional action or behavior [1]. Death by suicide accounted for more than a lakh reported cases from India [2]. World Health Organization (WHO) states that suicide is the second leading cause of death among those aged 15-29 years globally and hence is a major public health concern.

Suicidal rates are particularly higher in those whose first- and second-degree relatives are suicide completers and therefore, genetic factors account for the familial transmission of suicide. The estimated heritability of suicide is 30-50% [3]. Early life adversities are also considered important risk factors for lifetime suicidality. Life events such as childhood physical, emotional and sexual abuse, mental illness within the household, substance abuse within the household, and mother treated violently predisposes individuals to various mental health problems and also associated with increased suicidal tendencies [4]. Wetherall et al. reported low socioeconomic status as a risk factor contributing to suicide attempts [5].

Suicide is now thought to be a pro-inflammatory state and the role of inflammatory biomarkers for suicide attempters is under evaluation by various researchers [6-8]. Tumor necrotic factor-α (TNF-α) and interleukin-6 (IL-6) are inflammatory markers found altered in the blood of patients with schizophrenia and mood disorders [9]. C-reactive protein (CRP) is an acute-phase inflammatory protein synthesized by hepatic Kupffer cells causing damage to other cells and tissues by activation of the complement system. Several studies have shown that elevated high-sensitivity C-reactive protein (hsCRP) is a risk factor associated with physical and psychological morbidity for suicide and there is also an association between CRP and erythrocyte sedimentation rate (ESR) with the increased risk of suicide attempt [10-12]. Neutrophil-lymphocyte ratio (NLR) is a cost-effective, easy, and reproducible test. NLR represents two different but complementary immune pathways and is more informative than other white blood cells (WBC) parameters, such as IL-6 and TNF-α. NLR was higher in suicidal vs non-suicidal depressive patients and may be a biomarker of suicide attempts in major depressive disorder (MDD) [13,14].

The published data on hsCRP and NLR as markers of suicidal behavior is scanty. The present study was carried out with the primary objective of comparing the levels of inflammatory biomarkers (hsCRP and NLR) between suicide attempt survivors and healthy controls and further to evaluate the levels of those inflammatory biomarkers (hsCRP and NLR) with depression, stress, and anxiety symptom scores of suicide attempt survivors.

## Materials and methods

Study design and population

**Figure 1:**
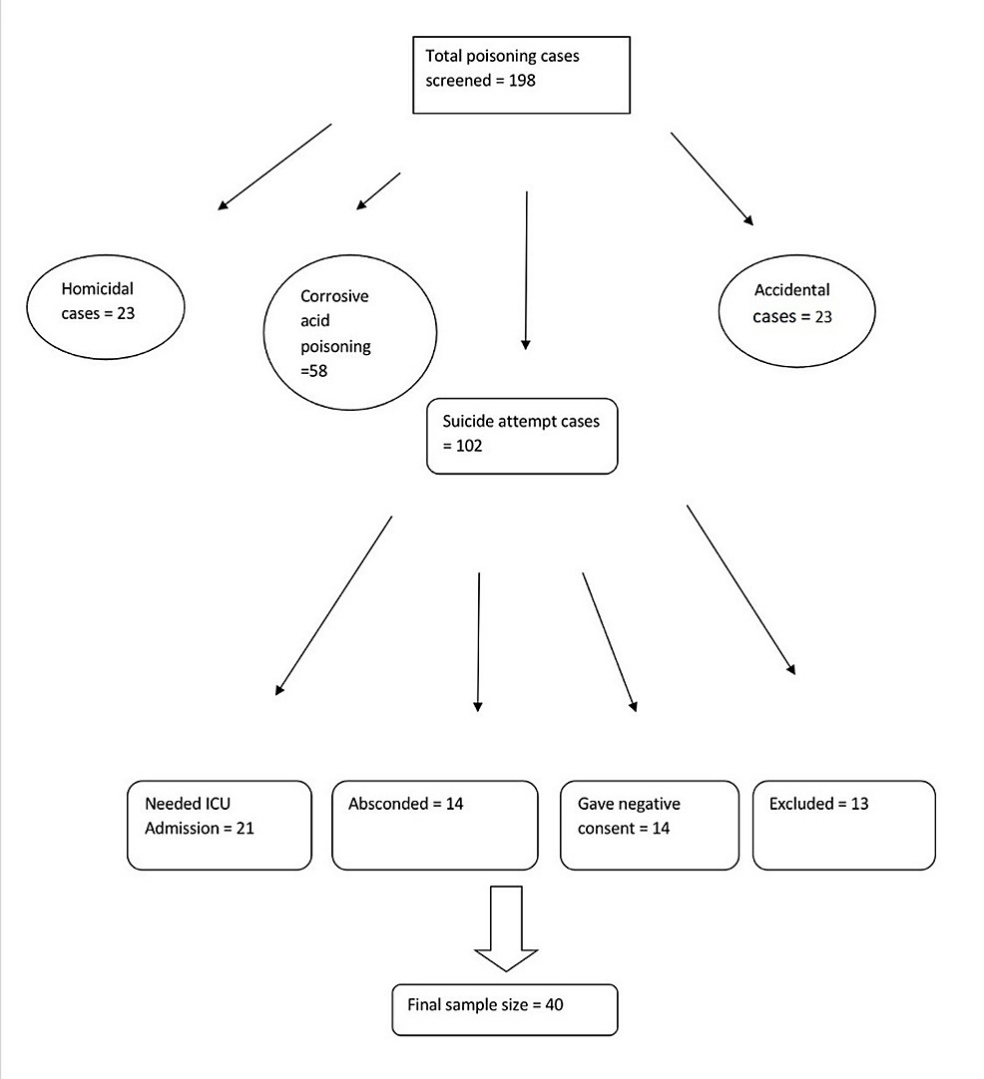
The image is illustrating the sample size selection (N=40)

The study sample comprising of poisoning suicide attempters visiting medicine emergency were recruited from November 2018 to April 2020 after thorough workup. The study was carried out jointly by the Department of Medicine & Psychiatry from the tertiary care, teaching hospital affiliated to a medical college of India. The current cross-sectional study consisted of 40 adults clinically diagnosed cases of poisoning, who had recently attempted suicide, aged between 18 years and 60 years, as shown in Figure 1. Forty age-matched healthy controls were recruited through advertisements posted on the notice boards of the hospital. The study included adult patients of poisoning who attempted suicide (18-60 years), both genders, after medico-legal evaluation. Poisoning suicide attempters with myocardial infarction, unstable angina, and any medical condition requiring urgent intervention, acute alcohol intoxication, patients of poisoning with altered mental status lasting for >48 hours, acute liver disease (serum aminotransferase >3 fold {>120 IU/l}), acute renal disease (serum creatinine levels >1.5 mg/dl), acute and chronic infectious diseases and patients requiring cardiopulmonary resuscitation (CPR) and intensive care unit (ICU) admission were excluded. The corrosive poisoning cases required immediate surgical intervention and hence, could not be taken up for the study. Cases with accidental consumption of poison and homicide were also excluded from the study as shown in Figure 1.

Data collection and evaluation

A detailed history including the chief complaints, history of presenting complaints, past history, personal history, family history, and dietary history detailed physical and mental state examination were carried out for all study subjects. Informed consent was obtained from all individual participants included in the study. Psychiatric questionnaires for assessment of depression, anxiety symptom scales, psychological morbidity, presumptive stressful life events scale, and cognitive functions were administered. Institutional ethics committee clearance was obtained before starting the research work.

Assessment of stress, anxiety, depression, and cognition

Beck’s suicide intent scale (SIS) was used to predict the suicide intent in a psychometric/measurable fashion in suicide attempters and a score of 15 or above has a specificity of 52% and sensitivity of 100% [15]. Presumptive stressful life events scale (PSLES) is an open-ended questionnaire in which study subjects rates the perceived event in their life on a score of zero to 100 where zero is considered no stress and 100 being the most stressful event in life [16]. Hospital anxiety and depression scale (HADS) is a self-rating scale used by patients to assess the severity of depression and anxiety. A score of seven or below is considered as normal, eight to 10 as mild, 11 to 14 as moderate, and 15 to 21 as severe for both anxiety and depression [17]. General health questionnaire 12- item (GHQ-12) (Hindi version) was used to assess the psychological health of the study subjects. The score items are scaled on Likert scale with scoring 0-0-1-1 for each of the four responses mentioned for each question. This tool is found to be sensitive and reliable and differentiates normal population from patient population indicating its high validity [18]. Hindi mental state examination (HMSE) is used as a cognitive screening tool and those with HMSE score more than 24 were recruited as study subjects for psychological evaluation as they are considered cognitively intact [19].

Assessment of inflammatory biomarkers

The neutrophil-lymphocyte ratio was calculated on five-part cell counter. hsCRP was measured by enzyme-linked immunosorbent assay (ELISA). hsCRP was done using a solid-phase sandwich ELISA. The kit was designed to measure human hsCRP in cell culture supernatants, serum and plasma. The present study utilized serum for hsCRP estimations and the estimations were conducted under the guidance of senior pathologist with more than twenty years of experience. The laboratory services of department of pathology are accredited by the national agencies.

Statistical analysis

Data were entered in MS-Excel and cleaned. Data were analyzed using SPSS 20.0 version (Armonk, NY: IBM Corp.). Categorical variables were expressed in percentages and quantitative variables as mean±SD. NLR and hsCRP were skewed in nature, therefore, a non-parametric Mann-Whitney U test was applied to test their independence and for subgroup analysis. Spearman rank correlation was used to find out correlation between inflammatory biomarkers and psychological morbidity. A p-value of <0.05 was considered significant.

## Results

The study group consisted of 40 cases, which had recently attempted suicide and aged between 18 years and 60 years, and 40 ages matched healthy controls. Table 1 compares the sociodemographic variables of cases and controls. The mean age of cases and controls was 28.28 and 28.98, respectively. There was no significant difference between the mean age of cases and controls. There were 32.5% males and 67.5% were females among the cases. There were 72.5% males and 27.5% were female among the controls. There was a female preponderance amongst the cases, while only one-third of cases were men. This was in contrast to the control group, where almost three-fourth of the individuals was male. There was a statistically significant difference between the cases and controls in terms of sex distribution (p=0.001). Almost half of the study participants had passed 12th class (Intermediate), while one-fourth of the study participants had at least a high school certificate. One study participant was illiterate.

**Table 1:** Sociodemographic profile of the study subjects (N=40) *p-Value<0.05 is considered significant.

Sociodemographic variables	Study subjects, n (%)	Controls, n (%)	p-Value
Gender			0.001*
Male	13 (32.5%)	29 (72.5%)	
Female	27 (67.5%)	11 (27.5%)	
Qualification			0.004*
Graduate/postgraduate	5 (12.5%)	21 (52.5%)	
Intermediate	19 (47.5%)	7 (17.5%)	
High school	10 (25%)	6 (15%)	
Middle school	3 (7.5%)	4 (10%)	
Primary school	2 (5%)	1 (2.5%)	
Illiterate	1 (2.5%)	1 (2.5%)	
Occupation			0.006*
Semi-professional/professional	11 (27.5%)	23 (57.5%)	
Clerical/shop owner	3 (7.5%)	7 (17.5%)	
Skilled worker	5 (12.5%)	5 (12.5%)	
Semi-skilled worker	9 (22.5%)	3 (7.5%)	
Unskilled worker	7 (17.5%)	1 (2.5%)	
Unemployed	5 (12.5%)	1 (2.5%)	

Hematological variables

The hsCRP was significantly higher in the case group as compared to the controls using both independent t-test (p=0.001) and Mann-Whitney U test (p=0.004). The NLR was higher among the case group, but the difference with controls was not statistically significant. Table 2 shows the comparison of hematological indices between the cases and controls.

**Table 2:** Comparison of hematological parameters, neutrophil-lymphocyte ratio, and hsCRP between cases and controls *p-Value<0.05 is considered significant. Hb: hemoglobin; RBC: red blood cells; MCV: mean corpuscular volume; MCH: mean corpuscular hemoglobin; MCHC: mean corpuscular hemoglobin concentration; WBC: white blood cells; NLR: neutrophil-lymphocyte ratio

Variables	Cases (mean±SD)	Controls (mean±SD)	Sig. (p-value)
Hb	11.63 (±2.20)	14.73 (±1.19)	<0.001*
RBC count	4.05 (±0.71)	5.10 (±0.59)	<0.001*
Hematocrit	36.40 (±6.25)	45.66 (±3.65)	<0.001*
MCV	91.35 (±12.99)	90.08 (±6.29)	0.0578
MCH	29.30 (±5.13)	29.09 (±2.34)	0.816
MCHC	32.18 (±2.83)	32.30 (±0.91)	0.791
WBC	8.53 (±2.88)	8.07 (±1.74)	0.393
Platelets	199.15 (±71.41)	210.95 (±52.81)	0.403
NLR	3.12 (±2.85)	2.38 (±0.76)	0.125
hsCRP	6090.31 (±4591.02)	3018 (±2909.52)	0.001*

There was a statistically significant difference in hemoglobin (p<0.001), RBC count (p<0.001), and hematocrit (p<0.001) between the cases and controls. All three were significantly higher among the control group. There was no difference between the cases and controls in the mean corpuscular volume (MCV), mean corpuscular hemoglobin (MCH), white blood cell count (WBC), and platelet count.

Analysis of NLR and hsCRP among anxious/depressed cases

There was a statistically significant difference (p<0.001) between HADS mean anxiety scores of cases (11.07±2.15) and controls (3.55±1.18). HADS depression mean scores were significantly different (p<0.001) between cases (10.75±2.46) and controls (3.52±0.96). Based on the HADS anxiety score, participants in the case group were labeled as anxious if their HADS (anxiety) score was more than 10, and as non-anxious, if the HADS (anxiety) score was equal to or less than 10. Similarly, the participants were labeled as depressed, if their HADS (depression) score was more than 10, and as non-depressed, if the HADS (anxiety) score was equal to as or less than 10.

In our study, four sub-groups of study subjects (n=40) were made using HADS scores, namely anxious-suicidal (n=24), depressed-suicidal (n=23), non-anxious suicidal (n=16), and non-depressed suicidal (n=17). The comparisons between anxious-suicidal vs non-anxious-suicidal (p=0.312 for NLR and 0.176 for hsCRP), depressed-suicidal vs non-depressed suicidal (p=0.256 for NLR and 0.753 for hsCRP), anxious-suicidal vs depressed-suicidal (p=0.928 for NLR and 0.704 for hsCRP), and non-anxious suicidal vs non-depressed suicidal (p=0.826 for NLR and 0.503 for hsCRP), and respective NLR and hsCRP values were done but no statistically significant difference was found in hsCRP and NLR values between the four sub-groups.

Further comparisons were made between the above four sub-groups namely, anxious-suicidal (n=24), depressed-suicidal (n=23), non-anxious suicidal (n=16), and non-depressed suicidal (n=17) and the control group as shown in Table 3. A statistically significant difference of the hsCRP (p=0.016) and NLR (p=0.029) between the depressed-suicidal vs controls was found. There is also a significant difference in the hsCRP between the anxious-suicidal vs controls (p=0.001). The hsCRP value of non-depressed vs controls also has a statistically significant difference with p-value=0.005.

**Table 3:** Comparisons of NLR and hsCRP between anxious (n=24), depressed (n=23), non-anxious (n=16), and non-depressed (n=17) suicidal subjects and controls (n=40) *p-Value<0.05 is considered significant. NLR: neutrophil-lymphocyte ratio; hsCRP: high-sensitivity C-reactive protein

Sub-categories	NLR	hsCRP
Anxious cases	3.22 (±3.08)	6934.77 (±4693.14)
Controls	2.38 (±0.76)	3018.04 (±2909.52)
p-Value	0.104	0.001*
Non-anxious cases	2.82 (±2.39)	4823.61 (±4263.39)
Controls	2.38 (±0.76)	3018.04 (±2909.52)
p-Value	0.298	0.073
Depressed cases	3.68 (±3.57)	6218.02 (±4771.16)
Controls	2.38 (±0.76)	3018.04 (±2909.52)
p-Value	0.029*	0.016*
Non-depressed cases	2.29 (±0.51)	5917.52 (±4474.47)
Controls	2.38 (±0.76)	3018.04 (±2909.52)
p-Value	0.657	0.005*

The correlation between hsCRP and NLR and any of the psychiatric test scores (HADS anxiety/depression, suicide intent scale, and Hindi GHQ and Mini Mental State Examination {MMSE}) was not statistically significant. PSLES was also used in this study and it was found that the mean (SD) of some life events is greater than the others. These particular items are responsible for psychological morbidity in the patients and primarily acting as a precipitating factor for suicide attempts. Mean (±SD) of those PSLES items which were significant (p<0.05) is as follows: 47.50 (±27.03) excessive alcohol use by the family member, 50 (±27.73) conflicts with in-laws, 50 (±29.42) family conflicts, and 50.63 (±32.76) marital conflicts respectively.

## Discussion

hsC-reactive protein, an inflammatory biomarker, was found to be altered in suicide attempters as compared to matched healthy volunteers. The current research further strengthens the role of inflammatory biomarkers in suicide attempt survivors (SAS) with or without prior history of psychiatric illness.

The prevalence figures of depressive symptoms in SAS were 57.5% and for anxiety symptoms were 60%. These high figures of psychiatric symptomatology highlight the need for early psychiatric evaluation in all suicide attempt survivors (SAS) as a part of consultation-liaison services. These findings have been supported by previous researchers.

Both the pharmacological as well as non-pharmacological psychiatric interventions were carried out for all suicide attempt survivors in the present work, so that further suicide attempts could be prevented because of low mood states. In a meta-analysis done to explore the relationships between marital status and suicide, the suicide risk was higher for non-married individuals <65years and higher for men than for women. Marriage acts as a protective factor, thereby providing emotional and social support plays a vital role in mitigating suicide risk [20]. In the present study, more than two-thirds of the cases were married at the time of recruitment and cases did show female preponderance with the mean age around 28 years. The possible explanation for higher marital figures (70%) in suicide attempt survivors in current study could be because of the exclusion criteria of extremes of age group in the present work. The study subjects (SAS) belonged to different cultural background subjected to different environmental conditions, 33% were newly or recently married females where adjustment problems were cited as the probable reason.

In a study conducted by Singh et al., a scale of stressful life events suitable for Indian population using open-ended questions relevant to Indian context was developed [16]. The present study applied presumptive stressful life events scale and calculated mean stress scores of 51 items. In SAS group, among 51 items of PSLES, those which ranked higher were excessive alcohol use by the family member, conflict with in-laws, family conflict, and marital conflict.

In the present study, hsCRP was significantly higher in SAS as compared to the control group. These findings were consistent with study conducted by Gibbs et al. [10]. Gibbs et al. compared hsCRP values of three sets of patients which were as follows: patients with suicide attempts, patients with suicide ideation, and inpatient psychiatric controls. They found hsCRP being higher in the patients with suicide attempts as compared to patients with suicidal ideation and inpatient psychiatric controls. They also observed a significant effect of depression in that depressed patient with higher hsCRP values were more predisposed to commit suicide attempts as compared to patients with no depression.

In a study conducted by Velasco et al., a sample of 538 Caucasian patients aged ≥18 years was recruited. They took patients who satisfied the diagnostic criteria of depression according to the Diagnostic and Statistical Manual of Mental disorders, Fifth Edition (DSM-5) [21]. The mean (±SD) NLR of 2.12 (±1.98) was observed in the total sample. The mean NLR of cases (recent suicide-attempters with MDD) and psychiatric controls (MDD patients with no history of suicide attempts) were 2.37 (±2.36) and 1.68 (±0.80), respectively with the p-value of 0.005. Therefore, there was a statistically significant difference between the two groups.

In the present study population, the mean NLR was 3.12 (±2.85) for the cases and 2.38 (±0.76) for the controls. The cases were further sub-divided into four sub-groups as depressive-suicidal (n=23), anxious-suicidal (n=24), non-depressive suicidal (n=17), and non-anxious-suicidal (n=16). Respective mean NLR in each sub-group was calculated and it was found that mean NLR of depressive-suicidal (suicide-attempter with depression) sub-group was 3.68 (±3.57) and that of controls (healthy subjects) was 2.38 (±0.76) with the p-value of 0.029, statistically significant. Thus, a statistically significant difference was found between mean NLR of depressive-suicide attempters and healthy controls. Hence, NLR serves as a potential biomarker for the depressive patients with suicidal attempt. Therefore, the findings of present study corroborate with the results of the study by Eknici, et al. and Velasco et al. [13,21]. Thus, NLR could be valuable, easily accessible, reproducible, and cost-effective biomarker to determine the risk of suicide in depressive patients.

In the present study, comparisons are made between depressed-suicidal patients and healthy controls with respect to their hsCRP and NLR values and we found a statistically significant difference between the hsCRP and NLR values of depressed-suicide attempters and healthy controls. Thus, indicating that hsCRP and NLR could be used as the predictors of suicide attempt in patients with a known history of depression.

This is in consistence with a study conducted by Orum et al., where they compared CBC parameters like total leukocyte count (TLC), neutrophil-lymphocyte ratio (NLR), and mean platelet volume (MPV) among the 38 violent suicide attempters and 38 non-violent suicide attempters [22]. TLC, NLR, and mean corpuscular volume (MCV) of suicide attempters are further compared with those of healthy controls. They found NLR to be significantly higher in violent suicide attempt group as compared to other two groups.

In the present study population, comparing hematological parameters like hemoglobin, RBC count, hematocrit, mean corpuscular volume (MCV), mean corpuscular hemoglobin (MCH), mean corpuscular hemoglobin concentration (MCHC), and platelet count of SAS with healthy controls revealed statistically significant difference in terms of hemoglobin, red blood cell (RBC), and hematocrit. Thus, indicating the fact that those with low levels of hemoglobin and poor state of nutrition are at increased risk of labile behavior, low mood, and suicide attempt.

Out of the 40 subjects, around eight subjects took pesticide (organophosphorus compounds), 14 subjects took rat kill (active ingredient is brodifacoum, warfarin-like action), four patients consumed All-out (prallethrin), two patients consumed celphos (aluminum phosphide), three patients took clonazepam tablets, one patient took alprazolam, one consumed lorazepam, one patient consumed nail thinner (butyl acetate and ethyl acetate), one patient consumed mercury tablets, one patient consumed few tablets of thyroxine, one patient consumed clonazepam tablets, and three patients consumed unknown substance which cannot be characterized. All suicide attempters were given primary care intervention in the casualty medicine department in the form of airway, breathing and circulation. Ryle’s tube lavage was done and if necessary, after stabilization, the psychiatric consultation was taken from the experienced psychiatrist and necessary psychosocial and medical interventions as per standard of care were carried out.

The results of present study showed that the hsCRP levels are higher in patients who have attempted suicide as compared to healthy controls. Therefore, hsCRP is an important indicator of both physiological and psychological parameters of increased stress. The differences in hemoglobin, RBC count, and hematocrit are also significant between suicide attempters and healthy controls, with fairly low levels seen in suicide attempters. Therefore, anemia is considered a risk factor for behavioral changes and suicidal risk. NLR and hsCRP levels were significantly higher in the depressive suicidal subjects as compared to the healthy controls. Thus, both NLR and hsCRP serve as the important peripheral biomarkers for evaluating the risk of suicide in depressive patients and early mitigation of these risk factors can prevent suicidal attempts and therefore, reduces the morbidity associated with depressive disorders.

Despite interesting findings, this study has some limitations. Firstly, it is a cross-sectional study that examined population at one point of time only. Secondly, most of the suicide attempters used self-poisoning as a method of self-harm. Thirdly, the cases and controls in the present study were not gender-matched. The future work requires larger sample size to study the variations in hsCRP values with reference to race, ethnicity, and gender.

The present study was conducted in patient population visiting tertiary care, teaching hospital which caters to different sections of the society with a sound methodology, using standardized tools with multidisciplinary standard of care provided to all study subjects. Further studies are required in this regard with bigger sample size and longitudinal follow-up.

## Conclusions

Both hsCRP and NLR have emerged as potential inflammatory biomarkers for depressive patients with suicidal attempts. Additionally, there may be a link between anemia and suicide risk in patients with depression.

Presumptive stressful life events such as alcohol use by family members, marital conflict, family conflict, and conflict with in-laws emerged as important contributory factors for attempted suicide. Timely assessment and early intervention of psychological morbidity using a multidisciplinary team approach may help reduce the risk of attempted suicide.
